# Effects of intratesticular injection of zinc-based solution in rats in combination with anti-inflammatory and analgesic drugs during chemical sterilization

**DOI:** 10.14202/vetworld.2018.649-656

**Published:** 2018-05-19

**Authors:** Simone Regina Barros de Macêdo, Luiz André Rodrigues de Lima, Sandra Maria de Torres, Vinícius Vasconcelos Gomes de Oliveira, Rosana Nogueira de Morais, Christina Alves Peixoto, Bruno Mendes Tenorio, Valdemiro Amaro da Silva Junior

**Affiliations:** 1 Department of Veterinary Medicine, Federal Rural University of Pernambuco, Recife, Brazil; 2 Department of Physiology, Federal University of Paraná, Curitiba, Paraná, Brazil; 3 Laboratory of Ultrastructure, Aggeu Magalhães Research Center (CPqAM), Recife, Pernambuco, Brazil; 4 Department of Morphology, Federal University of Paraíba, João Pessoa, Paraíba, Brazil

**Keywords:** analgesic, anti-inflammatory, contraception, testicular degeneration, zinc gluconate

## Abstract

**Aim:**

Chemical sterilization is a non-surgical method of contraception based on compounds injected into the testis to induce infertility. However, these injections can cause discomfort and pain able to impair the recovery of animals after this treatment. The objective of this study was to investigate if anti-inflammatories or pain relievers inhibited the sterilizing effect of zinc gluconate-based solution on the testis.

**Materials and Methods:**

Adult rats were treated in groups: G1 (control), G2 (dimethyl sulfoxide + dipyrone); G3 (dipyrone/zinc); G4 (dipyrone + celecoxib/zinc); G5 (dipyrone + meloxicam/zinc), and G6 (dipyrone + dexamethasone/zinc) in a single dose per day during 7 days. Animals were analyzed at 7, 15, and 30 days after treatments.

**Results:**

The zinc-induced a widespread testicular degeneration and decreased testosterone levels even in combination with anti-inflammatories or pain relievers. Testis, epididymis, prostate, and seminal vesicle had a weight reduction. The anti-inflammatory effect of dexamethasone interfered in the desired action of zinc gluconate in the 1^st^ 15 days and celecoxib up to 7 days.

**Conclusion:**

Meloxicam plus dipyrone did not impair the chemical sterilization based on zinc gluconate, and it can be used to reduce nociceptive effects in animals after chemical sterilization.

## Introduction

Dog and cat overpopulation is a serious problem in many cities around the world, characterized by a large number of ownerless animals living in public areas. These animals can be reservoirs or vectors for transmissible diseases dangerous to humans and economically valuable domestic species. Thus, it is necessary to develop more effective systems for birth control [1], and sterilization is an effective method to control animal populations [2]. Furthermore, many pet owners neuter their animals to reduce secondary sex characteristics such as mounting, aggression, and urine marking [3].

Chemical sterilization is a non-surgical method of contraception, in which chemical agents are injected into the testis, vas deferens, or epididymis, causing infertility by inducing azoospermia in males. It has been proposed as an alternative for the large-scale program for animals neutering. Among the chemical agents used in sterilization, zinc gluconate has been shown to be effective in young and adult animals [3-6].

The intratesticular injection of zinc solution produces an acute inflammatory process, with the probable generation of antibodies directed against testicular tissue [7]. As the action of zinc solution induces an inflammatory process, it is possible that some anti-inflammatory treatment disturbs their desired action as a sterilizer. Besides, zinc gluconate injection induces local edema, swelling, and necrotizing reactions in the scrotum of dogs [4,8].

Previous studies report discomfort after intratesticular injections, including Soto *et al*. [9] using Infertile^®^ (Rhobifarma, Hortolândia, SP, Brazil), as well as Wang [10] and Wang [11] using Neutersol^®^ (Pet Healthcare International, Columbia, MO, USA). According to Oliveira *et al*. [12], the efficacy of zinc gluconate (Testoblock^®^) as a chemical contraceptive in male dogs was not compromised in the presence of metamizole sodium (dipyrone). However, even though metamizole is able to induce a significant anti-nociceptive action, it does not have satisfactory anti-inflammatory response [13].

Therefore, the aim of this study was to determine whether administration of analgesic, or steroidal anti-inflammatory drug (SAID) and non-SAID (NSAID), is able to inhibit the effect of zinc gluconate solution on the testicular parenchyma of rats.

## Materials and Methods

### Ethical approval

The experimental protocol was approved by the Ethics Committee of the Federal University of Pernambuco (CEUA-UFPE 23076036668/2013-302013) in accordance with the basic principles for research using animals.

### Experimental design

Seventy-two male Wistar rats (*Rattus norvegicus*, var. Albinos) from the biotery (Department of Animal Morphology and Physiology of Federal Rural University of Pernambuco, Recife, Brazil) were randomly selected and kept in an environment with controlled temperature (23±2°C), humidity (60%), and 12 h light-dark cycle. Standard pellet food and water were provided *ad libitum*.

90-day-old rats were divided in groups according to the treatments: G1 Negative control/saline (n=12); G2 Positive control/dimethyl sulfoxide (DMSO) + dipyrone (n=12); G3 Infertile^®^ + dipyrone (n=12); G4 Infertile^®^ + dipyrone + celecoxib (n=12); G5 Infertile^®^ + dipyrone + meloxicam (n=12); and G6 Infertile^®^ + dipyrone + dexamethasone (n=12). Celecoxib was given orally (50 mg/kg) [14], dexamethasone was given intramuscularly (2 mg/kg) [15], and meloxicam (2 mg/kg) [16] and dipyrone (20 mg/kg) [17] were given by subcutaneous route.

### Intratesticular injections

The animals were anesthetized using ketamine 80 mg/kg and xylazine 15 mg/kg. Zinc gluconate-based solution was administered in both the testes of each animal parallel to the plane of testis in the dorsal cranial area of the testis, near the caput epididymis and the closest to the efferent duct [3]. The volume of solution injected was calculated according to the diameter of the testis as shown in Table-1. A caliper rule (Mitutoyo stainless, Andover, UK) was used to calculate the testicular diameter.

**Table-1:** Volume of zinc-based solution injected in the testes of rats according to testicular diameter.

Testicular diameter	Dose per testicle	

(mm)	mL	Zinc (mg)
8-9	0.3	3.9
10-11	0.4	5.2
11-12	0.5	6.6

Treatment with anti-inflammatory and analgesic drugs was done using a single dose per day during 7 days. Rats of each group were analyzed at 7, 15, and 30 days after testicular injection with zinc gluconate-based solution.

### Histopathological analysis of the testes

After euthanasia (Thiopental 150 mg/kg), animals were subjected to an intracardiac perfusion using a NaCl 0.9% solution plus heparin (50 IU/l) for 15 min [18]. Thereafter, rats were again perfused with a paraformaldehyde 4% in phosphate buffer (0.1 mol/L and pH 7.3) for 10 min. The epididymis, testis, and accessories glands were removed, dissected, and weighted using a balance (BEL MARK 500, Monza, Italy).

Fragments of the testes were cut into 2-mm thickness, refixed in paraformaldehyde solution for 4 h, and routinely embedded in paraffin. Histological sections (4 µm) were stained with hematoxylin/eosin and analyzed morphologically as previously described by de Siqueira Bringel *et al*. [19].

### Electron microscopy

Testicular fragments were processed for electron microscopy according to Cavalcanti *et al*. [20]. Briefly, the fragments of the testes were fixed overnight in a solution containing 2.5% glutaraldehyde and 4% paraformaldehyde in 0.1 M cacodylate buffer. The samples were post-fixed in a solution containing 1% osmium tetroxide, 2 mM CaCl_2_, and 0.8% potassium ferricyanide in 0.1 M cacodylate buffer (pH 7.2). Testis tissue was dehydrated in acetone and embedded in SPIN-PON (Embed 812). Ultrathin sections were stained with uranyl acetate (3%) and lead citrate and imaged and photographed in a transmission electron microscope (Jeol JEM 100 CX, Tokyo, Japan).

### Plasma testosterone

Blood samples were obtained through puncture of the vein cava and centrifuged, and the plasma was kept at −20°C until analysis [21]. Plasma samples were analyzed by enzyme-immunoassay using a polyclonal anti-testosterone antibody (enzyme-linked immunosorbent assay) with absorbance reading at 405 nm, according to Brown *et al*. [22]. All samples were read in duplicate, with both intra- and inter-assay coefficients of variation of <10%.

### Statistical analysis

The Shapiro–Wilk test was used to verify the tendency to normality of the results. Depending on the normality, we used analysis of variance with the Tukey-Kramer *post-hoc* test (parametric) or the test of Kruskal–Wallis with Dunn’s *post-hoc* test (nonparametric). Data were expressed as mean, (±) standard deviation, and p<0.05.

## Results

### Weight of reproductive organs

Table-2 shows testicular, epididymal, prostate, and seminal gland weight (g). At 15 days, there was a reduction in testis weight in G4 (dipyrone + celecoxib) compared to G1 (control). At 30 days, animals in G3 (dipyrone) and G4 (dipyrone + celecoxib) had the testis weight decreased compared to G1 (control). There was a reduction in epididymis weight in G4 (dipyrone + celecoxib) and G6 (dipyrone + dexamethasone) after 30 days. Further, after 30 days, rats in G5 (dipyrone + meloxicam) showed a reduction in prostate weight. Similarly, the rats treated with dipyrone + dexamethasone (G6) showed a reduction in prostate weight. After 15 days, the seminal gland weight also showed a reduction in G3 (dipyrone) and G4 (dipyrone + celecoxib).

**Table-2:** Weight of testis, epididymis, prostate, and seminal gland (g) of Wistar rats treated with intratesticular injection of zinc gluconate and evaluated at 7, 15, and 30 days after treatment. G1 (saline), G2 (DMSO), G3 (zinc+dipyrone), G4 (zinc+celecoxib+dipyrone), G5 (zinc+meloxicam+dipyrone), and G6 (zinc+dexamethasone+dipyrone).

Organ	G1	G2	G3	G4	G5	G6
Testis (days)						
7	1.2±0.2	0.9±0.2	1.3±0.1	1.2±0.2	1.2±0.1	1.6±0.7
15	1.8±0.5^a^	1.2±0.2^ab^	1.0±0.2^ab^	0.7±0.04^b^	1.0±0.3^ab^	1.1±0.2^ab^
30	1.6±0.3^a^	0.7±0.2^ab^	0.4±0.1^b^	0.5±0.06^b^	1.0±0.4^ab^	0.6±0.1^ab^
Epididymis (days)						
7	0.6±0.1	0.7±0.1	0.6±0.4	0.6±0.1	0.4±0.2	0.7±0.3
15	1.8±1.0	0.4±0.1	0.5±0.06	0.7±0.1	0.4±0.2	0.4±0.1
30	1.1±0.2^a^	0.5±0.1^ab^	0.4±0.2^ab^	0.3±0.1^b^	0.5±0.1^ab^	0.2±0.09^b^
Prostate (days)						
7	0.6±0.1	1.1±0.2	0.2±0.2	0.6±0.1	0.4±0.2	0.4±0.1
15	0.7±0.2	0.7±0.3	0.4±0.08	0.2±0.1	0.5±0.3	0.3±0.2
30	1.6±0.5^a^	0.9±0.1^ab^	0.4±0.06^ab^	0.7±0.2^ab^	0.1±0.1^b^	0.2±0.0^b^
Seminal gland (days)						
7	1.0±0.04	1.1±0.3	0.4±0.2	0.4±0.2	0.6±0.2	0.7±0.3
15	1.7±0.4^a^	1.1±0.5^ab^	0.6±0.4^b^	0.3±0.05^b^	0.7±0.5^ab^	0.8±0.4^ab^
30	1.7±0.4	1.9±0.4	0.2±0.05	0.2±0.2	0.7±0.6	0.6±0.3

Different letters in the same line are equivalent to statistically significant difference (p<0.05), DMSO=Dimethyl sulfoxide

### Plasma testosterone

Table-3 shows the concentrations of testosterone in plasma. Note the absence of difference in testosterone between G1 and G2 indicates that DMSO by itself was not enough to reduce testosterone levels. Paradoxically, the rats treated with an intratesticular injection of zinc gluconate showed a reduction in testosterone levels of 74%, 66%, and 53% at 7, 15, and 30 days, respectively. It is noteworthy that all groups treated with zinc gluconate greatly reduced the plasma testosterone (G3, G4, G5, and G6) even in the presence of dipyrone, celecoxib, meloxicam, or dexamethasone. Despite this large reduction, some of these results were not statistically significant due to the wide dispersion of the data.

**Table-3:** Plasma testosterone (ng/mL) of Wistar rats treated with intratesticular injection of zinc gluconate and evaluated at 7, 15, and 30 days after treatment. G1 (saline), G2 (DMSO), G3 (zinc+dipyrone), G4 (zinc+celecoxib+dipyrone), G5 (zinc+meloxicam+dipyrone), and G6 (zinc+dexamethasone+dipyrone).

Time	G1	G2	G3	G4	G5	G6
7 days	1.11±0.3^a^	1.36±1.18^a^	0.21±0.03^b^	0.26±0.09^ab^	0.28±0.14^ab^	0.28±0.8^ab^
15 days	1.03±0.3	1.60±0.78	0.23±0.03	0.41±0.30	0.49±0.53	0.23±0.07
30 days	1.88±0.28^ab^	2.09±0.34^b^	0.31±0.24^ab^	0.9±1.07^ab^	0.24±0.05^ab^	0.21±0.08^a^

Different letters in the same line are equivalent to statistically significant difference (p<0.05), DMSO=Dimethyl sulfoxide

### Histopathology of the testes

**Figure-1:**
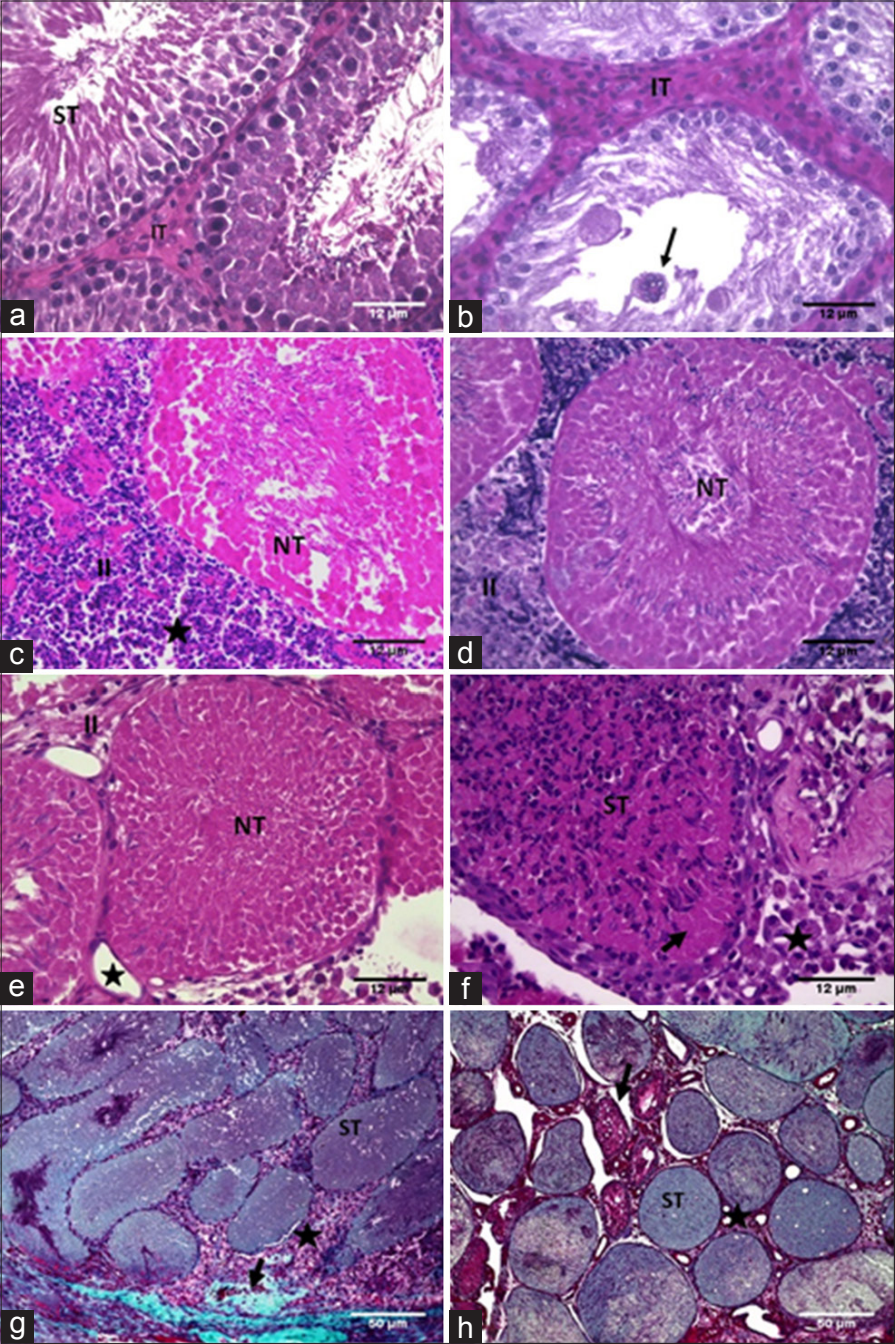
Photomicrographs of testis (hematoxylin-eosin) of adult rats submitted to intratesticular injection of a zinc-based solution and treated with different anti-inflammatory drugs for 7 days and evaluated at 15 and 30 days. (a) Seminiferous tubule (ST) at Stage VII of the seminiferous epithelium cycle and intertubular tissue (IT) in the control group. (b) Animal treated only with dimethyl sulfoxide (DMSO). Note the increased IT and syncytial giant cells (arrow). (c) Necrotic seminiferous tubule (NT) and increased IT (II) with inflammatory infiltrate and cellular debris (star) in an animal treated only with dipyrone. (d) Necrotic seminiferous tubule (NT) and increased IT with inflammatory cells and cellular debris (II) in the celecoxib-treated group. (e) Animal treated with meloxicam, note necrotic seminiferous tubule (NT), increased IT (II) and blood vessels (star). (f) Animal treated with dexamethasone. Note ST within polymorphonuclear leukocyte infiltrates (ST), germ cell necrosis (arrow), and IT with macrophages (star). (g) Animal treated with dipyrone stained using Gomori Trichrome. ST within necrotic germ cells (ST), IT filled by macrophages (star) and fibrosis (arrow). (h) Animal treated only with DMSO stained using Gomori Trichrome. ST within necrotic germ cells (ST) and IT without inflammatory cells (star). Note atrophic ST (arrow).

Analyses of histopathological lesions in the testes are shown in Table-4 and Figure-1. Testicular parenchyma in the control group (G1) at 7, 15, and 30 days showed normal seminiferous tubules (STs), including germ cells lines as spermatocytes I in leptotene and pachytene stage, round and elongated spermatids, as well as large quantities of sperm (Figure-1a).

**Table-4:** Histopathological lesions in the testes of Wistar rats treated with intratesticular injection of zinc gluconate and evaluated at 7, 15 and 30 days after treatment. G1 (saline), G2 (DMSO), G3 (zinc+dipyrone), G4 (zinc+celecoxib+dipyrone), G5 (zinc+meloxicam+dipyrone) and G6 (zinc+dexamethasone+dipyrone).

Lesion	7 days							15 days							30 days						

	Groups						AF (RF%)	Groups						AF (RF%)	Groups						AF (RF%)

	G1	G2	G3	G4	G5	G6		G1	G2	G3	G4	G5	G6		G1	G2	G3	G4	G5	G6
Thickening of tunica albuginea	-	4 (100%)	4 (100%)	4 (100%)	4 (100%)	4 (100%)	24 (100%)	-	3 (75%)	2 (50%)	2 (50%)	4 (100%)	4 (100%)	15 (62%)	-	3 (75%)	1 (25%)	4 (50%)	4 (50%)	-	12 (50%)
Fibroblast proliferation	-	4 (100%)	-	-	4 (100%)	-	8 (33%)	-	4 (100%)	2 (50%)	1 (25%)	4 (100%)	-	11 (45%)	-	4 (100%)	4 (100%)	2 (50%)	4 (100%)	3 (75%)	13 (54%)
Inflammation	-	4 (100%)	4 (100%)	2 (50%)	4 (100%)	1 (25%)	15 (62%)	-	3 (75%)	3 (75%)	2 (50%)	4 (100%)	1 (25%)	13 (54%)	-	1 (25%)	2 (50%)	4 (50%)	4 (50%)	4 (100%)	15 (62%)
Tubular necrosis	-	4 (100%)	4 (100%)	4 (100%)	4 (100%)	4 (100%)	24 (100%)	-	3 (75%)	4 (100%)	4 (100%)	4 (100%)	4 (100%)	19 (79%)	-	4 (100%)	4 (50%)	3 (75%)	2 (50%)	4 (100%)	17 (70%)
Dystrophic calcification	-	2 (50%)	4 (100%)	-	4 (100%)	3 (75%)	13 (54%)	-	-	2 (50%)	-	4 (100%)	3 (75%)	9 (37%)	-	1	2(50%)	-	-	2 (50%)	5 (20%)
Neovasculari zation	-	-	-	-	-	-	-	-	-	2 (50%)	-	-	-	2 (8%)	-	-	-	-	2 (50%)	-	2 (8%)
Tubular degeneration	-	3 (75%)	4 (100%)	4 (100%)	4 (100%)	3 (75%)	18 (75%)	-	4 (100%)	4 (100%)	3 (75%)	4 (100%)	3 (75%)	18 (75%)	-	4 (100%)	3 (75%)	4 (100%)	-	4 (100%)	14 (58%)
Congestion	-	2 (50%)	3 (75%)	-	4 (100%)	-	9 (37%)	-	-	-	2 (50%)	4 (100%)	-	6 (25%)	-	2 (50%)	-	1 (25%)	1 (25%)	-	4 (16%)
Subcapsular edema	-	1 (25%)	-	-	1 (25%)	-	2 (8%)	-	-	-	-	1 (25%)	-	1 (4%)	-	1 (25%)	-	-	-	-	1 (4%)

AF=Absolute frequency, RF=Relative frequency, DMSO=Dimethyl sulfoxide

G2, G3, G4, G5, and G6 at 7, 15, and 30 days after intratesticular injection of zinc gluconate showed high frequency of syncytial giant cells (Figure-1b), inflammation (Figure-1c), germ cell degeneration and necrosis (Figure-1d), increased intertubular tissue (IT) (Figures-1e and f), thickening of the tunica albuginea, dystrophic calcification, congestion, tubular degeneration with vacuolated Sertoli cells, and IT showing macrophages (Figures-1g and h). Some areas in the IT showed necrotic Leydig cells. Little fibroblast proliferation was observed 7 days after zinc treatment, but this was more frequent in G2 and G5. At 15 and 30 days, this proliferation was very frequent in other groups except for the G4 and G6. During all experimental period, there were little subcapsular edema and neovascularization.

G3 showed the higher severity of the pathological lesions than G2; therefore, zinc gluconate is critical to produce infertility compared only with DMSO. G4 showed fewer lesions and inflammation up to 7 days and G6 up to 15 days. Dexamethasone + dipyrone (G6) interfered with the desired action of zinc gluconate in the 1^st^ 15 days. Treatment with celecoxib + dipyrone (G4) little interfered with zinc gluconate action, especially at the beginning of treatment. Meloxicam + dipyrone (G5) showed the better results.

### Electron microscopy

**Figure-2:**
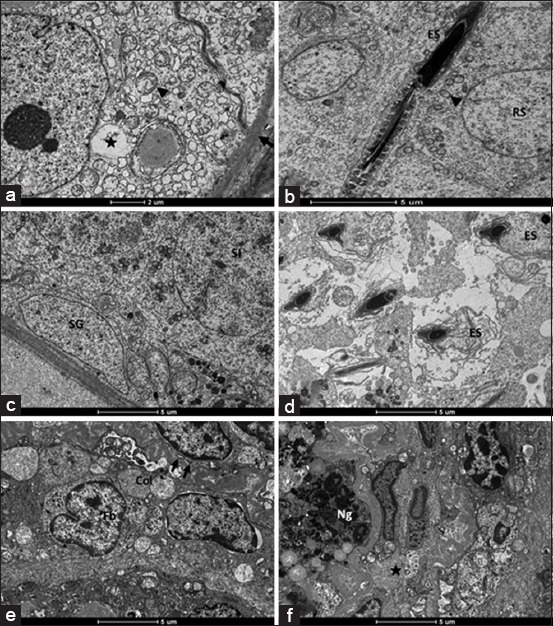
Transmission electron micrograph of testis in adult rats treated with intratesticular injection of a zinc-based solution and treated with different anti-inflammatory drugs (dipyrone + dexamethasone: (a and b); dipyrone + celecoxib: (c and d); dipyrone + DMSO: (e and f). (a) Note basal membrane thickness (arrow), Sertoli cell vacuolization (star) and mitochondria damage (arrowhead). (b) Observe elongated spermatid (ES) and round spermatids showing well-preserved structure but with mitochondria damage (arrowhead). (c) Note spermatogonia and spermatocytes I showing a well-preserved cell structure. (d) Swollen ES detached from Sertoli cell crypt. (e) Active fibroblast, collagen deposition in the intertubular tissue and thickening of basal membrane in an atrophied seminiferous tubule (arrow). (f) Intertubular fibrosis (star) and necrotic germ cell (Ng).

The ultrastructural evaluation of G2, G3, and G5 showed widespread damage in the testis tissue. Corroborating the histopathological analysis, G4 and G6 showed lesser ultrastructural lesions compared to the other groups treated with zinc gluconate. These ultrastructural lesions were characteristic of testicular cells suffering toxic damage. We observed basal membrane thickness, Sertoli cell vacuolization, mitochondria damage, swollen and sloughed germ cells, necrotic germ cell, activated fibroblasts, and large collagen deposits (Figure-2). The experimental groups also showed some areas with well-preserved cellular structure alternating with large areas of ultrastructural lesions. These lesions were more frequent in G3 (zinc + dipyrone) and G5 (zinc + dipyrone + meloxicam).

## Discussion

Intratesticular injection of a sterilizing agent promotes an immune response caused by rupture of the testicular blood barrier with subsequent inflammation and release of testicular antigens [10]. This inflammatory reaction inhibits spermatogenesis once cytokines, reactive oxygen species, and glucocorticoids have deleterious effects on the seminiferous epithelium [2,23,24].

Testicular weight has a positive relationship with testicular function and spermatogenesis, as well as this weight reduction suggests a loss of germ cells [25]. The reduction in epididymis weight also indicates decreased spermatogenesis and hence reduction in epididymis content [26]. The reduced weights of reproductive organs observed in this study suggest that anti-inflammatories did not affect the weight reduction triggered by zinc gluconate-based solution.

The activity of the seminal gland and prostate in the adult is closely related with the level of androgens, especially testicular testosterone, and they may reflect changes in the animal’s endocrine status or testicular function [27]. The reduction in plasma testosterone levels was directly related to the reduction in the prostate and seminal vesicle weight.

Intratesticular treatment with zinc gluconate in dogs also induced a reduction in levels of testosterone 40-80% [5]. Testosterone is related to sexual behavior in males; therefore, one of the sterilization goals is to reduce the inconvenient sexual behaviors [28,29]. In this study, we observed that chemical sterilization using zinc gluconate was able to reduce the activity of Leydig cells and testosterone levels even after the association with the pain reliever dipyrone and anti-inflammatories celecoxib, meloxicam, or dexamethasone.

The principle of chemical sterilization by intratesticular injection of zinc-based solution is to produce irreversible changes in testicular tissue, effectively impairing spermatogenesis [5]. Oliveira *et al*. [5] observed pathological lesions in the seminiferous epithelium with destruction of spermatocytes, spermatids, and absence of spermatozoa, resulting in the sterility of dogs. Atrophied STs, basal membrane thickening, and intertubular fibrosis were also observed by Fagundes *et al*. [3]. On the other hand, a zinc gluconate-based solution tested in young adult male dogs did not cause permanent azoospermia 12 months after intratesticular application, but the sperm showed low concentration and motility; this study also showed testicular degeneration, decreased number of germ cells, STs atrophy, and loss of Sertoli cells [30].

According to Oliveira *et al*. [12], the pain reliever dipyrone was not able to suppress the local inflammatory response caused by the intratesticular injection of zinc gluconate. Therefore, choosing a suitable anti-inflammatory therapy to associate with dipyrone to reduce the adverse effects of chemical sterilization with zinc is very important to increase the acceptance of this type of procedure.

The present study showed that dipyrone + meloxicam in therapeutical association did not change the sterilizing property of zinc gluconate. The pathological lesions in this group were very severe even with great analgesic, anti-inflammatory, and antipyretic potential of meloxicam via inhibition of COX-2 [31]. Therefore, meloxicam did not reduce the ability of zinc gluconate to cause testicular degeneration.

The COX-2 inhibitor celecoxib seems to delay the onset of the inflammatory reaction induced by the zinc gluconate. According to Hilário *et al*. [32], COX-2 inhibitors (celecoxib and rofecoxib) were more efficient than sodium diclofenac (ibuprofen) to reduce inflammatory reaction induced in the dental pulp of rats. This was also observed in this study when compared to the group only treated with dipyrone.

The orchitis due to trauma or testicular torsion is characterized by an acute inflammatory reaction that can be minimized by NSAIDs and non-COX-2 NSAIDs [33]. In general, the COX-2 inhibitors are usually indicated to musculoskeletal diseases such as arthritis, bone disease, and arthrosis. The use of COX-2 inhibitors after orchitis chemically induced was not found in the previous literature. The present study demonstrated that COX-2 inhibitor did not suppress the sterilizing effect of zinc gluconate. Thus, these anti-inflammatories can be considered as an antalgic and antipyretic to avoid discomfort and improve the animal welfare after chemical sterilization.

Dexamethasone inhibits the production of prostaglandins, leukotrienes, and thromboxanes that are important to chemotaxis and activation of the inflammatory process [34]. Dexamethasone treatment postponed the testicular inflammation to 15 days after chemical sterilization in this study. This great anti-inflammatory effect of dexamethasone can interfere with the desired action of zinc gluconate, and it is not recommended in the sterilization protocol. DMSO has anti-inflammatory, analgesic, and vasodilatation activity, improves the microcirculation, and affects platelet aggregation [35]. In this study, DMSO by itself exerted deleterious effects on the testicular parenchyma. On the other side, plasma testosterone levels showed that DMSO did not affect Leydig cells. Therefore, lesions caused by DMSO possibly can be reversible, and DMSO alone should not be used as a sterilizing agent.

## Conclusion

Intratesticular injection of zinc gluconate was effective to reduce plasma testosterone and damage testicular tissue, proving to be effective as a neuter agent even after use of anti-inflammatory and analgesic therapy. However, the anti-inflammatory effect of celecoxib and dexamethasone interfered with the desired action of zinc gluconate during the 1^st^ 7 and 15 days, respectively. The association meloxicam + dipyrone can be the anti-inflammatory and analgesic therapy of choice in the chemical sterilization protocol using zinc.

## Authors’ Contributions

SRBM, LARL: Performed the animals treatments; SMT: Prepared the histological sections; SMT, VVGO and BMT: Performed the histopathological analysis; RNM: Performed the plasma testosterone analysis; CAP: Performed the electron microscopy; BMT: Drafted this manuscript; VASJ: Oversaw all stages of the present study and drafted and revised this manuscript. All authors read and approved the final manuscript.
